# The validation of the visual screening tool for anxiety disorders and depression in hypertension and/or diabetes

**DOI:** 10.4102/phcfm.v10i1.1722

**Published:** 2018-11-14

**Authors:** Zimbini Ogle, Liezl Koen, Dana J.H. Niehaus

**Affiliations:** 1 Department of Psychiatry, Stellenbosch University, South Africa; 2 Stikland Psychiatric Hospital, Bellville, South Africa

## Abstract

**Background:**

Depression and anxiety disorders remain poorly detected at primary health care, particularly in patients with hypertension and/or diabetes. A visual screening tool for anxiety disorders and depression (VISTAD) has been developed, but not validated.

**Aim:**

To validate the VISTAD in primary health care participants diagnosed with hypertension and/or diabetes.

**Setting:**

Participants were recruited from five primary health care centres in the Eastern Cape, South Africa (urban, peri-urban and rural).

**Methods:**

The study used a cross-sectional study design to validate the VISTAD. The VISTAD was validated against the International Neuropsychiatric Interview (M.I.N.I) using field testing. A demographic questionnaire was used to collect data on socio-economic variables.

**Results:**

Sixty-nine (87%) females and 10 (13%) males with a mean age of 49 (SD 8.6844) participated in the study. Fifty black people (63%), 16 mixed race people (20%) and 13 white people (16%) participated in the study. The majority of the participants (77%) did not complete high school. The area under curve score (AUC) for the VISTAD in screening for depression was 0.91, and for anxiety disorders, 0.87 post-traumatic stress disorder, 0.87 panic disorder, 0.85 social phobia, 0.88 agoraphobia, and 0.83 generalised anxiety disorder revealing acceptable psychometric properties.

**Conclusion:**

The use of the VISTAD as a screening tool at primary health care in people living with hypertension and/or diabetes is recommended. The VISTAD could, therefore, play a key role in the prevention and early treatment of individuals diagnosed with hypertension and/or diabetes across cultures and levels of education. The VISTAD needs to be validated in a large population representative of primary care patients diagnosed with hypertension and/or diabetes.

## Background

Current literature demonstrates that hypertension and diabetes have emerged as a major medical and public burden globally.^1,2,3,4^ South Africa is burdened with a high prevalence of hypertension and diabetes. There are 2.3 million people living with diabetes in South Africa,^5^ and 30% of the adult population is living with hypertension.^6^ Furthermore, hypertension and diabetes account for 17 million visits to health facilities in South Africa every year.^1^

The diabetes and hypertension burden is further complicated by the increasingly high co-morbidity with depression and anxiety disorders. Kumar and Clark^7^ argue that chronic diseases have psychological sequelae; however, these remain largely undetected and untreated at primary health care.^8,9,10^ Patients may be aware of their emotional state; however, they may be unable to describe accurately their subjective experience, according to Aitken.^11^ In addition, inadequate levels of mental health literacy, for both health care workers^12^ and patients, have been well established in research.^13^

When clinicians do attempt to screen for mental disorders in patients with hypertension and/or diabetes, primary health care facilities are faced with barriers such as insufficient human and material resources, lack of assessment instruments that can be appropriately applied to the diverse range of cultural and language groupings in South Africa, and communication difficulties which lead to misunderstandings, misdiagnosis and/or inappropriate treatment. In addition to the above-mentioned barriers, pencil and paper tests are often not available or have to be read aloud to illiterate patients.^14^ Availability of appropriate tools at primary health care could therefore contribute to the quality of detection and management of mental disorders, particularly in developing countries. Visual screening tools for depression and anxiety disorders could possibly circumvent the challenges posed by cultural, language, educational and time factors. This has been shown by Akena et al.^15^ who developed a visual screening tool for depression in patients living with HIV and/or AIDS in Uganda. However, the screening tool developed by Akena et al.^15^ does not screen for anxiety disorders. Screening for depression, according to Katon et al.,^16^ should also include anxiety disorders as these often coexist in patients living with chronic physical conditions.

The use of pictures in aiding patients to describe emotions and thoughts has been well established in psychology. Psychological tests, referred to as projectives, such as the thematic apperception test (TAT),^17^ make use of drawings in order to allow access to unconscious thoughts, emotional life and internal dynamics – revealing hidden materials that clients are unable to disclose or unwilling to disclose. Ogle, Koen and Niehaus developed a visual screening tool for anxiety disorders and depression (VISTAD) using drawings based on the hospital anxiety and depression scale (HADS). The visual screening tool is referred to as the VISTAD. It includes depression items such as sleep disturbance, feeling miserable and sad, appetite and feeling life is not worth living, as well as anxiety items such as feeling frightened or having panic feelings for no reason, feeling frightened when going out of the house alone, getting palpitations or sensations ‘butterflies’ in stomach or chest, more irritable than usual and worrying thoughts. The VISTAD, however, has not been validated for use in primary health care.

The aim of the study was to validate the VISTAD as a screening tool for use in primary health care in individuals diagnosed with hypertension and/or diabetes and as a tool that can be used effectively in a time- and resource-constrained environment and with people with low levels of education.

## Methods

### Study design

The study used a cross-sectional study design for validating the newly developed VISTAD.

### Setting

The study was conducted in five primary health care centres in the Eastern Cape, South Africa. These primary health care centres provide health care services to two urban areas, KwaMagxaki, a predominantly black suburb, and Algoa Park, a mixed suburb, mixed race and white population, in Port Elizabeth; one peri-urban area in Uitenhage, predominantly black population; and two rural communities, KwaNonqubela, a black community, and Wentzel Park, a mixed race community in Alexandria, Eastern Cape.

### Sampling strategy

Purposive sampling was utilised to recruit participants who were able to provide informed consent. Individuals were recruited while they were at the settings for their routine scheduled visit. Individuals between the ages of 18 and 60, diagnosed with diabetes and/or hypertension, were eligible for the study. Individuals known to have visual and hearing impairments, and intellectual disability were excluded from the study.

The principal researcher explained the purpose of the study and requested individuals who were interested in participating in the study to indicate their interest. Those who indicated an interest or wanted to get more information were interviewed in a private setting and provided with more details on the study.

### Data collection

A demographic questionnaire was utilised to gather information about gender, age, race, marital status, level of education, employment status, family income and medical conditions. A short structured diagnostic interview, the International Neuropsychiatric Interview (M.I.N.I),^18^ was used as a gold standard in the validation of the VISTAD. It covers 17 Axis I disorders that include mood, anxiety, substance use, psychotic and eating disorders, and it also has a suicidality module and one Axis-II disorder, antisocial personality disorder.^18^ Sheehan et al.^18^ found the M.I.N.I to be a reliable and valid diagnostic tool, and it has been used in South African studies.^19,20,21,22^ The development of the VISTAD is discussed in detail in Ogle, Koen and Niehaus.^23^ In the VISTAD, a score of 1 is allocated when a participant endorses an abnormal state drawing and 0 when none of the abnormal state symptoms are endorsed. The maximum score that could be obtained on the VISTAD is 10.

### Data analysis

Descriptive statistics were used to describe demographic data. The M.I.N.I was used to categorise cases and non-cases of depression (major depressive episode) and anxiety disorders, that is, agoraphobia, generalised anxiety disorder (GAD), panic disorder, post-traumatic stress disorder (PTSD) and social phobia, excluding obsessive–compulsive disorder. The impact of education, employment and gender on the performance of VISTAD was investigated. Coefficient of correlation (coef.) indicated the correlation between the above-mentioned variables. The sensitivity and specificity and likelihood ratios (LR) were estimated. Sensitivity and specificity was calculated based on leave-one-out cross validation. Cut-off scores were informed by high specificity scores and moderate sensitivity scores. With a high specificity, the truly positives represent the mental disorder being screened for and not another condition, such as diabetes or hypertension.

The plot of sensitivity versus specificity is referred to as the receiver operating characteristics (ROC).^24^ The area under the curve (AUC) which is an effective measure of diagnostic test accuracy^24^ was used for interpretations of the data. An AUC value of 0.50–0.70 is considered low accuracy, 0.70–0.90 is considered moderate accuracy and 0.90 is considered high accuracy.^25^ Linear discriminant analysis was used to predict the disorder outcomes with the VISTAD drawing outcomes as predictors. All data analyses were done with STATA, version 14.

### Ethical considerations

Ethical approval was granted by the University of Stellenbosch’s Faculty of Medicine and Health Sciences Human Research Ethics Committee (Reference number: S14/11/262). Permission to conduct the study at the primary care centres was obtained from the Eastern Cape Department of Health, South Africa

## Results

### Demographics

Eighty-one participants from primary health care participated in the validation of the VISTAD. All the participants consented and participated in this study. However, out of the 81 participants, one declined to continue with completing the interview reporting that the VISTAD depicted his life, and it was painful to look at the images.

In this study, we used demographic data of 79 participants as there was missing information from two participants. The majority of the participants were females, 69 (87%), with 10 (13%) males. Race distribution on the basis of gender showed that there were 43 black females, 15 mixed race females and 11 white females, and seven black males, two white males and one mixed race male. The mean age of the participants was 49, with standard deviation 8.6844 and minimum age 22 and maximum 60. Socio-demographic variables and related factors are set out in Table 1.

**TABLE 1:** Demographic and clinical characteristics of participants (*N* = 79).

Variables	*n*	(%)
**Gender**		
Female	69	87
Male	10	13
**Language**		
isiXhosa	48	61
Afrikaans	29	37
English	1	1
Shona	1	1
**Race**		
Black people	50	63
Mixed race	16	20
White people	13	17
**Education**		
No education	2	3
Primary level of education (Grades 1–6)	25	32
Senior level of education (Grades 7–11)	33	42
Matric (Grade 12)	15	19
Post-matric qualification	4	5
**Employment**		
Yes	21	27
No	58	73
**Hypertension or diabetes**		
Hypertension only	47	59
Diabetes only	1	1
Hypertension and diabetes	31	39
**Other medical conditions**		
Yes	43	54
No	36	46
**Existing mental disorder**		
Yes	10	13
No	69	87
**M.I.N.I. diagnosis**		
Depression	26	32
Panic disorder	32	40
Agoraphobia	8	10
Social phobia	8	10
Post-traumatic stress disorder	27	33
Generalised anxiety disorder	14	17

### Accuracy of the visual screening tool for anxiety disorders and depression

**FIGURE 1:**
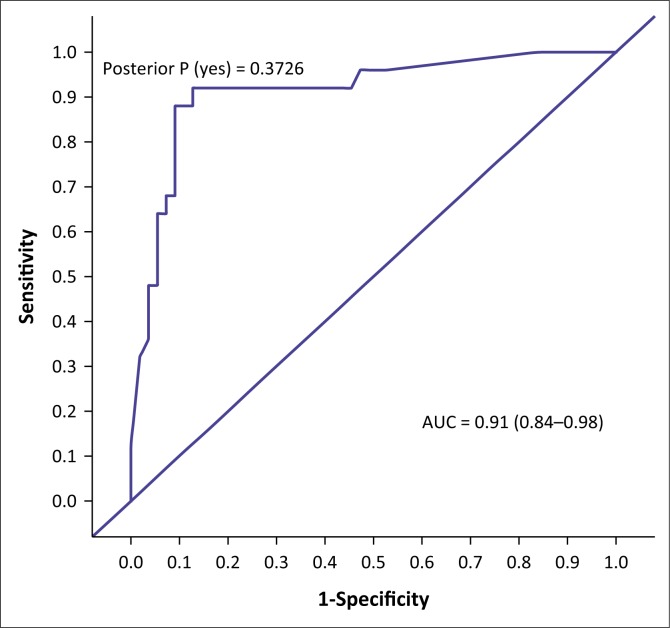
Receiver operating curves of the visual screening tool for anxiety disorders and depression for depression.

**FIGURE 2:**
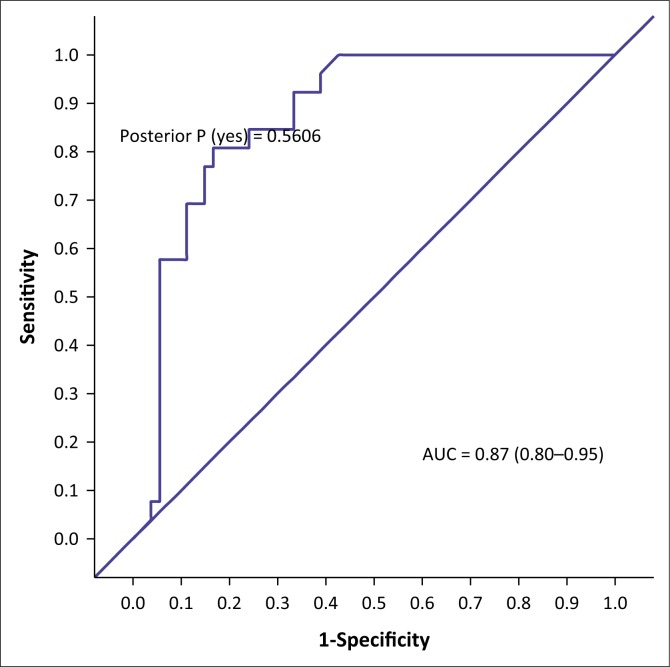
Receiver operating curves of the visual screening tool for anxiety disorders and depression for post-traumatic stress disorder.

**FIGURE 3:**
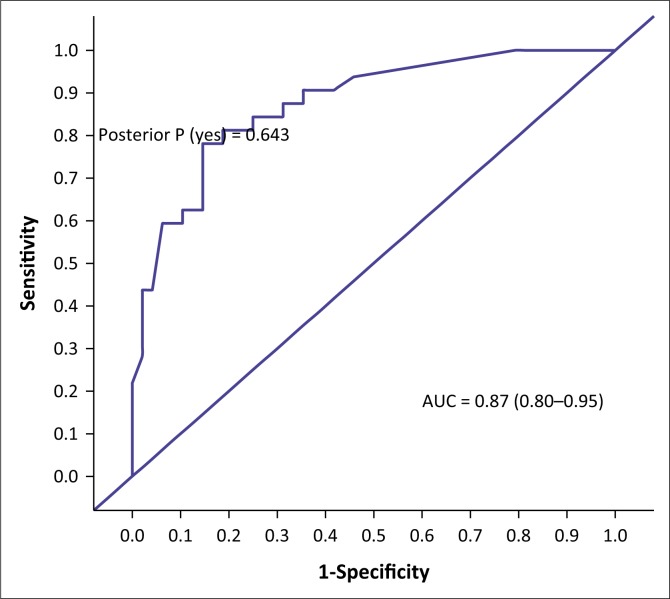
Receiver operating curves of the visual screening tool for anxiety disorders and depression for panic disorder.

**FIGURE 4:**
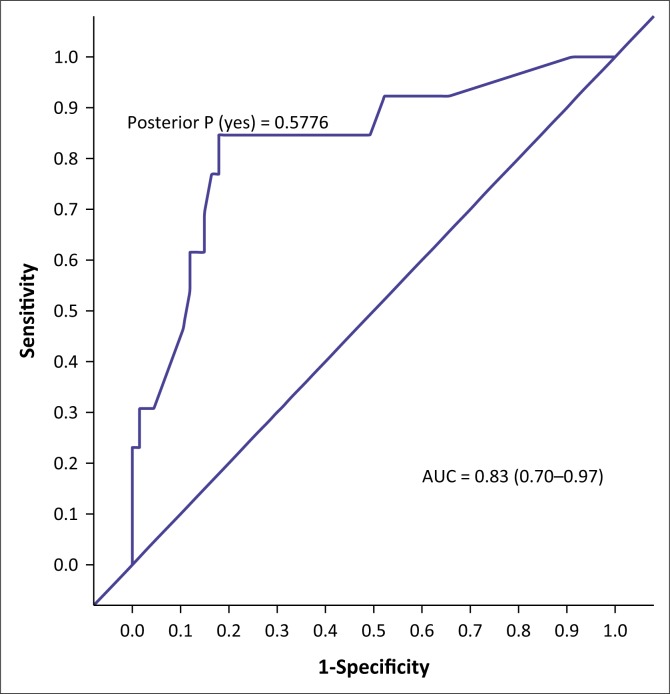
Receiver operating curves of the visual screening tool for anxiety disorders and depression for generalised anxiety disorder.

**FIGURE 5:**
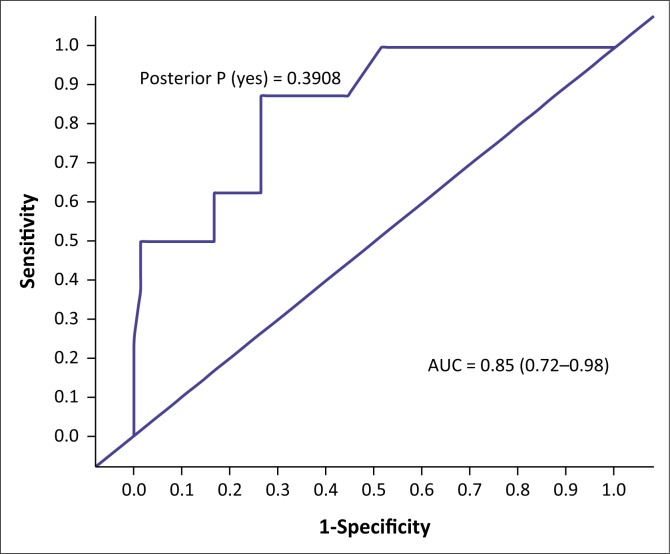
Receiver operating curves of the visual screening tool for anxiety disorders and depression for social phobia.

**FIGURE 6:**
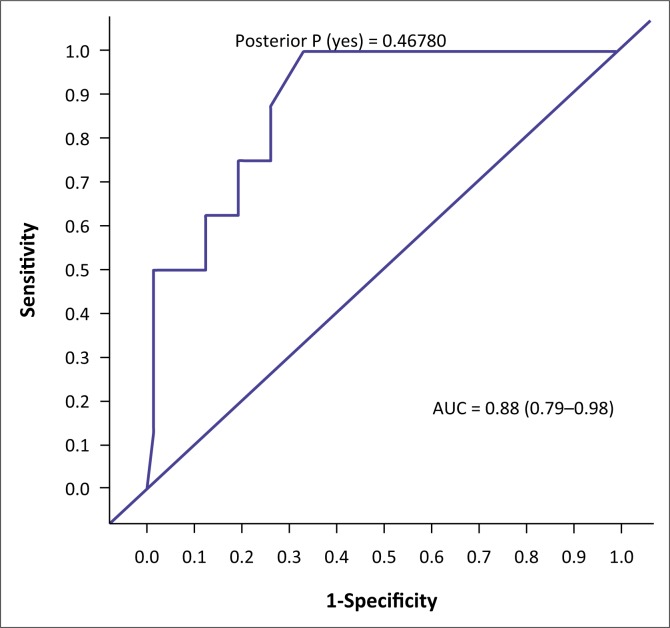
Receiver operating curves of the visual screening tool for anxiety disorders and depression for agoraphobia.

The AUC determined the accuracy of the VISTAD. The AUC in screening for depression is shown in Figure 1, PTSD in Figure 2, panic disorder in Figure 3, GAD in Figure 4, social phobia in Figure 5 and agoraphobia in Figure 6. The AUC score for depression shows a high accuracy of 0.91.

The best cut-off scores were based on high specificity and moderate sensitivity. At a cut-off score of 6, the specificity was 90.91, and sensitivity was 72.00%, with 85% correctly classified cases. The specificity was 90.91 and sensitivity was 60.00%, with 81.25% correctly classified cases at a cut-off score of 7.

The area under curve for PTSD was 0.87. At a cut-off point of 6 for PTSD, there were 68% correctly classified cases with a specificity of 72.22% and a sensitivity of 37%. Similar to PTSD, the area under curve for panic disorder was 0.87, which indicated moderate accuracy. The specificity of the VISTAD at a cut-off point of 6 was 81.25%, with a sensitivity of 43.75%.

Agoraphobia had a specificity of 73.61%. Social phobia had a specificity of 72.22% at a cut-off score of 6. Generalised anxiety disorder had a specificity of 74.63%, with 70% correctly classified cases.

Fundamental to the validation of the VISTAD is the investigation of whether education levels, socio-economic status and gender have an impact on the performance of the VISTAD. Table 2 presents the impact of level of education, gender and employment status on the performance of the VISTAD.

**TABLE 2:** Impact of education, gender and employment status on the performance of the visual screening tool for anxiety disorders and depression.

Variable	Coef.	SE	*T*	*p* > |*t*|	95% CI
**Level of education**					
Primary	−0.7505694	3.557147	−0.21	0.833	−7.841612–6.340473
Senior	−0.6239653	3.507459	−0.18	0.859	−7.615957–6.368026
Matric	−2.326809	3.619307	−0.64	0.522	−9.541764–4.888147
Post-matric	0.0663401	4.089943	0.02	0.987	−8.086812–8.219493
**Gender**					
Male	−3.715155	1.795807	−2.07	0.042	−7.295031– -0.1352794
**Employment**					
Yes	0.4993359	1.26012	0.40	0.693	−2.012668 –3.01134
_cons	4.808826	3.425357	1.40	0.165	−2.019499–11.63715
/sigma	4.344278	0.4488455			3.449521–5.239035

CI, confidence interval; SE, standard error; coef., coefficient of correlation; cons, coefficient estimate; sigma, standard deviation.

## Discussion

### Findings

In this study, we validated the VISTAD against the M.I.N.I. at primary health care in participants diagnosed with hypertension and/or diabetes. The VISTAD demonstrated high accuracy in detecting depression in participants with hypertension and/or diabetes. The AUC of the VSTAD was 0.91 in screening for depression. At a cut-off score of 6, the VISTAD had satisfactory accuracy in classifying cases. This is similar to that of the HADS which the VISTAD is based on. At a cut-off score of 7, the HADS depression subscale provided the best balance between a sensitivity of 0.86 and a specificity of 0.81 in cancer patients.^26^ In our study, the best balance was at a cut-off score of 6 with a sensitivity of 0.72 and a specificity of 0.91, with 85% of the cases classified correctly. The accuracy of the VISTAD is also similar to that of other widely used traditional screening tools, such as the Patient Health Questionnaire (PHQ), with an AUC of 0.88 at a higher cut-off score in patients with type II diabetes and/or coronary heart disease in primary care,^27^ and the Kessler Scale (K10) for either anxiety or depression^28^ and CES-D, K-10 and PHQ-9 and with AUC ranging from 0.82 to 0.96^15^ in people living with HIV.

The VISTAD had a higher AUC score compared to the recently validated WHO-5 for use in screening for depression in adults with diabetes. The AUC scores for WHO-5 in the validation study by Halliday et al.^29^ ranged between 0.85 and 0.88, which demonstrated that the WHO-5 has moderate accuracy in screening for depression. This demonstrates that the VISTAD is a valuable tool for detecting depression. The optimal cut-off score chosen for depression was 6 in this study. This is in line with the current practice of selecting higher cut-off scores to indicate the presence of depression.^28,29^ Similar to screening for depression, the optimal cut-off score chosen for anxiety disorders was 6. The AUC for anxiety disorders was in the moderate range. This indicates that the accuracy of the VISTAD was better for depression than for anxiety. This is consistent with findings made in the meta-analysis conducted by Vodemaier and Millman^26^ on other screening tools. Previous research has also established a similar pattern. For example, GAD 7 showed lower sensitivity and specificity levels when compared to screening tools for depression.^28^ Makanjuola et al.^30^ also reported lower AUC values in Nigeria for K-6 and the General Health Questionnaire (GHQ-12). K-6 and GHQ-12 are sensitive and specific screening tools widely used and recommended for use in the screening for depression and/or anxiety disorders in primary health care and community samples.

Newly developed screening tools have also been recommended for use in patients with chronic medical conditions. A Ugandan study recommended the use of a visual screening tool for depression.^15^ This was based on its accuracy in detecting depression in people living with HIV. However, a study by Puertas^31^ did not recommend the use of visual screening tools as it found the FACES test to have low accuracy. The FACES test, according to Puertas, is a visual analogue scale representation of mood, consisting of seven graded faces from happiest mood to saddest mood. Akena^15^ argues that screening tools, such as the FACES test, have often depended on a single facial picture depicting emotions ranging from a happy face to an extremely sad face. Participants with lower literacy levels struggle to comprehend the FACES screening tool according to Puertas^31^ Previous research has established that education has an impact on people’s ability to comprehend and complete screening tools.^32,33^ Some, according to Snaith,^34^ are ashamed and pretend to answer questions and respond in a haphazard manner. This study demonstrated that education had no impact on the participants’ ability to comprehend and complete the VISTAD. The findings on the education and performance of the VISTAD are consistent with previous research by Akena et al.^15^ Furthermore, the socio-economic status had no impact on the participants’ ability to understand and complete the VISTAD. The majority of participants in this study were of low socio-economic status.

Based on the M.I.N.I, we noted a high prevalence of common mental disorders in this study. For example, depression had a prevalence of 32%. This is consistent with the findings of Jacob and Kostev’s study,^35^ which reported a prevalence of 33.7% in women and 26.8% in men diagnosed with diabetes. Cols-Sagarra et al.^36^ reported a higher prevalence of 43.4% in women with diabetes at primary health care. A prevalence of 31.4% was reported in a study conducted in rural and urban parts of the Eastern Cape, South Africa,^37^ and this finding is consistent with the prevalence observed in our study.

Anxiety disorders such as panic disorder and PTSD had a high prevalence, with panic disorder at 40% and PTSD at 33%. Other studies have found a significantly higher prevalence of panic disorder in hypertensive patients.^38^ Also, in primary care, studies have observed a prevalence of PTSD ranging from 2.0% to 39.1% in hypertensive patients.^39^ Post-traumatic stress disorder, according to Seedat^40^ is among the most prevalent compared to other anxiety disorders in terms of lifetime and 12-month prevalence rates documented in epidemiological studies. The number of participants who had a positive diagnosis of a common mental disorder was high. This observation is consistent with previous research findings.^39,41,42^ Data on larger samples are needed to determine the prevalence of depression and anxiety disorders in primary care patients with hypertension and/or diabetes.

### Strengths and limitations

This is the first study to develop and validate a visual screening tool for both depression and anxiety disorders in primary health care participants diagnosed with hypertension and/or diabetes in South Africa. Furthermore, the use of the M.I.N.I in the study was not limited to the depression module, and the anxiety disorder module was also administered, except for obsessive–compulsive disorder module. The administration of the M.I.N.I depression module only might artificially inflate the extent of the correlations as some participants with depression might have been better diagnosed with another disorder, such as anxiety disorders.^15^ Also, this could lead to a false accuracy of the visual screening tool. The strength of the study was enhanced by the inclusion of five different primary health care sites, which serve urban, peri-urban and rural population.

Because of the small size of the sample, we cannot generalise the findings of the study to all individuals attending primary health care. Thus, future research is needed to validate the VISTAD in a large primary health care population. Furthermore, the use of the VISTAD in primary health care with speech impairments and intellectual impairments needs further investigation. A further limitation is that this study was informed by the DSM-IV nosology. For example, PTSD is no longer described as an anxiety disorder in the current DSM-V, but as a trauma- and stressor-related disorder.

## Conclusion

The visual screening tool is referred to as the VISTAD. The VISTAD is accurate in detecting depression and anxiety disorders in primary health care participants diagnosed with diabetes and/or hypertension. The use of the VISTAD is recommended as a screening tool for depression and anxiety disorders at primary care level in patients with hypertension and/or diabetes. However, the VISTAD needs to be validated in a large population of primary care patients diagnosed with hypertension and/or diabetes.
