# Erratum: The validation of the visual screening tool for anxiety disorders and depression in hypertension and/or diabetes

**DOI:** 10.4102/phcfm.v11i1.2008

**Published:** 2019-08-29

**Authors:** Zimbini Ogle, Liezl Koen, Dana J.H. Niehaus

**Affiliations:** 1 Department of Psychiatry, Stellenbosch University, South Africa; 2 Stikland Psychiatric Hospital, Bellville, South Africa

In the version of this article published earlier, the funding information was omitted. The note is hereby provided as: ‘The authors acknowledge the funding support provided by the Maternal Mental Health Fund, Rural Health Fund, National Research Foundation and the National Health Scholars Programme’. This correction does not alter the study’s findings of significance or overall interpretation of the study results. The publisher apologises for any inconvenience caused.

